# Peer-led recovery groups for people with psychosis in South Africa (PRIZE): protocol for a randomised controlled feasibility trial

**DOI:** 10.1186/s40814-022-01232-8

**Published:** 2023-02-01

**Authors:** Laura Asher, Bongwekazi Rapiya, Julie Repper, Tarylee Reddy, Bronwyn Myers, Charlotte Hanlon, Inge Petersen, Carrie Brooke-Sumner

**Affiliations:** 1 Academic Unit of Lifespan and Population Health, School of Medicine, University of Nottingham, Nottingham, UK; 2 Alcohol, Tobacco and Other Drug Research Unit, South African Medical Research Council, Francie Van Zijl Drive, Parow Valley, Cape Town, 7501 South Africa; 3 Implementing Recovery through Organisational Change, Nottingham, UK; 4 Biostatistics Research Unit, South African Medical Research Council, Durban, South Africa; 5 Curtin enAble Institute, Curtin University, Perth, Western Australia 6151 Australia; 6 Department of Psychiatry and Mental Health, University of Cape Town, J-Block, Groote Schuur Hospital, Observatory, Cape Town, South Africa; 7 Centre for Global Mental Health, Health Service and Population Research Department, Institute of Psychiatry, Psychology and Neuroscience, King’s College London, London, UK; 8 Department of Psychiatry, College of Health Sciences, School of Medicine, Addis Ababa University, Addis Ababa, Ethiopia; 9 Centre for Rural Health, College of Health Sciences, University of KwaZulu-Natal, Durban, South Africa

**Keywords:** Schizophrenia, Psychotic disorders, Sub-Saharan Africa, Developing countries, Psychosocial intervention, Psychiatric rehabilitation, Self-help groups, Community mental health services, Peer-led, Recovery

## Abstract

**Background:**

The available care for people with psychosis in South Africa is inadequate to support personal recovery. Group peer support interventions are a promising approach to foster recovery, but little is known about the preferences of service users, or the practical application of this care model, in low- and middle-income countries (LMIC). This study aims to assess the acceptability and feasibility of integrating peer-led recovery groups for people with psychosis and their caregivers in South Africa into existing systems of care, and to determine key parameters in preparation for a definitive trial.

**Methods:**

The study is set in Nelson Mandela Bay Metropolitan district of the Eastern Cape Province, South Africa. The design is an individually randomised parallel group feasibility trial comparing recovery groups in addition to treatment as usual (TAU) with TAU alone in a 1:1 allocation ratio. We aim to recruit 100 isiXhosa-speaking people with psychosis and 100 linked caregivers. TAU comprises anti-psychotic medication-focused outpatient care. The intervention arm will comprise seven recovery groups, including service users and caregiver participants. Recovery groups will be delivered in two phases: a 2-month phase facilitated by an auxiliary social worker, then a 3-month peer-led phase. We will use mixed methods to evaluate the process and outcomes of the study. Intervention acceptability and feasibility (primary outcomes) will be assessed at 5 months post-intervention start using qualitative data collected from service users, caregivers, and auxiliary social workers, along with quantitative process indicators. Facilitator competence will be assessed with the GroupACT observational rating tool. Trial procedures will be assessed, including recruitment and retention rates, contamination, and validity of quantitative outcome measures. To explore potential effectiveness, quantitative outcome data (functioning, unmet needs, personal recovery, internalised stigma, health service use, medication adherence, and caregiver burden) will be collected at baseline, 2 months, and 5 months post-intervention start.

**Discussion:**

This study will contribute to the sparse evidence on the acceptability and feasibility of peer-led and recovery-oriented interventions for people with psychosis in LMIC when integrated into existing care systems. Results from this feasibility trial will inform preparations for a definitive trial and subsequent larger-scale implementation.

**Trial registration:**

Pan-African Clinical Trials Register PACTR202202482587686. Registered on 28 February 2022. https://pactr.samrc.ac.za/TrialDisplay.aspx?TrialID=21496.

**Supplementary Information:**

The online version contains supplementary material available at 10.1186/s40814-022-01232-8.

## Background

Globally, people with psychosis experience high levels of unmet needs, including social and economic hardships and human rights violations [1–3]. In South Africa, whilst most people with psychosis have access to primary care clinic-based outpatient services (primarily free provision of anti-psychotic medication) and inpatient care, there is limited community-based support for personal recovery. As a consequence, there are high readmission rates following discharge from hospital [4]. Recovery has been described as, “a deeply personal, unique process of changing ones’ attitude, values, feelings, goals, skills and/or roles” and “a way of living a satisfying, hopeful and contributing life even within the limitations caused by illness” [5]. The meaning of recovery, along with how we can best support and measure it, is shaped by cultural and social context. For example, it is proposed that family support has greater influence on recovery in low- and middle-income countries (LMICs) compared to high-income countries (HICs). Likewise, social connectedness and interdependence may be more pertinent indicators of recovery in LMICs [6].

Community-based psychosocial support offers benefits in terms of reducing symptom severity and improving functioning in people with psychosis [7–9]. Task-sharing, that is the delivery of circumscribed aspects of healthcare by less specialised workers with specialist support, is championed to increase coverage of community-based care. For real-world implementation to be successful, it is essential that task-shared approaches are integrated into existing health and social care systems. Furthermore, irrespective of the delivery agent, care should be person-centered [10], in order to be capable of fostering personal recovery. Highly structured psychosocial interventions may be favoured in LMIC settings to ensure non-specialist workers can become interventionists after only brief training within a task-sharing approach [11], but there are concerns that this approach could be to the detriment of truly person-centred care [10].

Peer support mental health interventions are provided by people with lived experience in one-to-one or group formats and can encompass emotional support, advocacy, and activities to promote social inclusion. There is a strong emphasis on personal recovery, by using a non-judgmental approach to address the issues of importance to service users. As experts by experience, peers are ideally placed to understand and support people with mental health conditions [12]. The World Health Organization (WHO) promotes peer support workers as a means of expanding coverage of community-based mental health care [13]. Peer support work is one modality of task-sharing, so it may be a useful strategy in settings with few specialist mental health professionals. Peer-led recovery groups have potential to achieve a person-centred approach, in which participants’ own needs and priorities are front and centre. The response to those needs is not preordained, but rather is a dynamic response arising from the diverse and real-life experiences of other group members. Such groups might also build peers’ self-confidence through meeting others, managing similar struggles, and reducing self-stigmatisation through positive role modelling. Peer support groups might also provide a good fit in LMIC settings where family and socially oriented mechanisms of recovery are prominent [6].

Whilst there is little evidence to date that peer support approaches provide greater benefits than usual care in reducing hospital readmission or relapse [14–17], there is some evidence that group peer support interventions are effective in supporting personal recovery in people with schizophrenia, depression, and bipolar disorder [17]. However, there is an absence of high-quality evidence for peer support approaches for people with psychosis in LMIC [18]. With the exception of one Chinese trial, all studies included in four recent systematic reviews were conducted in HIC [14, 16, 17, 19]. Peer-led mental health support groups are nevertheless used successfully in some LMIC. For example, the Users and Survivors of Psychiatry in Kenya supports a thriving network of service user-initiated and led peer support groups, with an emphasis on exercising respect for legal capacity (including treatment decisions) and advocacy activities [12]. The acceptability of the peer support model may be influenced by socio-cultural norms. For example, in Chile, where high importance is placed on professional and social hierarchies, service users seem less likely to accept support from an individual deemed not hierarchically superior [20]. Despite this emerging evidence, there has been little formal evaluation of the acceptability and feasibility of this model in LMIC [21–23]. In South Africa, recovery groups are not available as part of existing health and social care structures; evidence of the feasibility, acceptability, and potential benefits of these groups are needed to inform future investment in these kinds of services. To address this gap, we developed the peer-led recovery groups for people with psychosis in South Africa (PRIZE) intervention. This involved an 18-month formative phase that included scoping work; in-depth interviews with service users, caregivers, and service providers; and collaborator workshops (to be reported separately). Building on our model of group psychosocial rehabilitation previously piloted in South Africa’s North West Province [24, 25], we designed a task-shared model delivered in two phases: an auxiliary social worker (ASW)-facilitated phase followed by a peer-facilitated phase. Both emphasise peer involvement in group delivery, underpinned by a strong recovery orientation.

The overall aim of this randomised feasibility trial is to assess the acceptability and feasibility of the PRIZE intervention for people with psychosis in South Africa, and to determine key parameters in preparation for a definitive trial. The objectives, which span a process evaluation, the assessment of trial procedures, and a preliminary outcome evaluation are:

### Process evaluation

- 1.a. To assess the acceptability and feasibility of the intervention from the perspective of service users, caregivers and service providers (primary outcomes)
- 1.b. To assess the skills and competence of ASW and peer facilitators

### Assessment of trial procedures

- 2.a. To establish the validity of quantitative outcome measures of recovery, unmet needs, and functioning
- 2.b. To assess trial procedures including the acceptability and feasibility of fidelity measures, recruitment and retention rates, and the suitability of an individually randomised design
- 2.c. To synthesise outcome data to inform the sample size of a definitive trial

### Preliminary outcome evaluation

- 3.a. To explore the potential effectiveness of recovery groups plus treatment as usual (TAU) compared to TAU alone in terms of service user functioning, personal recovery, unmet needs, internalised stigma, health service use, relapse, medication adherence, and alcohol consumption; and caregiver burden

## Methods

### Setting

The study site is in the Eastern Cape province, which has the lowest Gross Domestic Product per capita of all nine provinces in South Africa. We will work in the Nelson Mandela Bay Metropolitan district (population 1,152,115) that includes the city of Gqeberha, two small towns and agricultural areas. There are 41 primary health care facilities (“clinics”) and eight hospitals (including a psychiatric hospital). Eight of the clinics provide mental health care delivered by psychiatric nurses. Available care includes free medication for people with psychosis living in the community. The clinics do not provide psychosocial support for people with psychosis. Seven of these eight clinics serve a predominantly isiXhosa-speaking population (the target population for the study). These seven study sites are in low-income areas with a concentration of low education levels, unemployment, and poor health outcomes.

### Study design

**Fig. 1:**
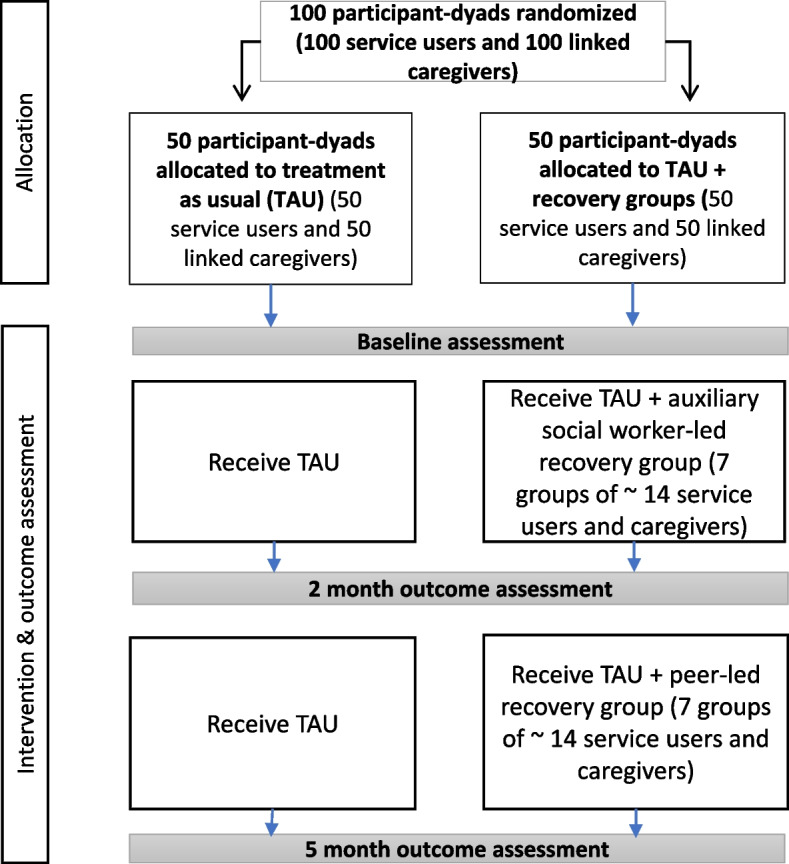
PRIZE study flow chart

The design is an individually randomised parallel group feasibility trial comparing recovery groups in addition to TAU compared to TAU alone in a 1:1 allocation ratio (see Fig. 1). We will use mixed methods to address our study objectives. Data will be collected at baseline, 2 months, and 5 months post-intervention start.

### Participants

**Fig. 2:**
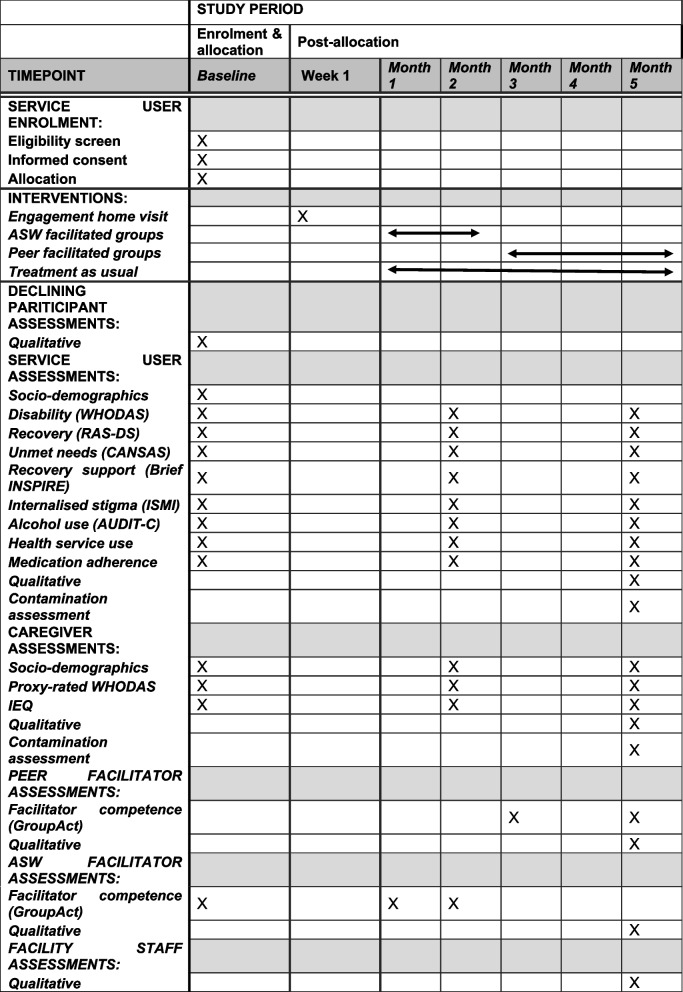
PRIZE participant timeline

Trial participants are service users and caregivers. Service user eligibility criteria are as follows: (i) clinical diagnosis of psychosis associated with enduring disability, including schizophrenia, schizoaffective disorder or dual diagnosis of schizophrenia, and alcohol use or other substance use disorder; (ii) ≥18 years old; (iii) speak isiXhosa; (iv) have capacity to give informed consent to study participation; and (v) plan to stay in the area for the next 6 months. Caregiver eligibility criteria are as follows: (i) primary caregiver for a participating service user; (ii) ≥18 years old; (iii) speak isiXhosa; and (iv) plan to stay in the area for the next 6 months. Additional participants for the process evaluation are recovery group facilitators and supervisors (ASWs and a social worker), and TAU service providers (psychiatric nurses and clinic managers). A subset of potential participants who meet the eligibility criteria but who decline to participate in the trial will be invited to participate in a brief qualitative interview. The participant timeline is presented in Fig. 2.

#### Recruitment

We estimate that a minimum of 15 people with psychosis access care at each participating clinic. Information leaflets and posters will be used to provide initial sensitization to the study in the clinic waiting areas. At these clinics, during all regular scheduled appointments for people with psychosis taking place during the recruitment period, psychiatric nurses will provide initial brief information about the study and ask for verbal consent for a PRIZE staff member to speak with them or contact them. The nurse will use a script to clarify that the person has no obligation to participate and that non-participation will not affect their care. Service users who agree will be directed to speak with an assessor immediately following their clinic appointment. The assessor will give initial verbal information on the study to the service user in isiXhosa and complete a screening checklist for eligibility criteria (except capacity to consent). A home visit will then be arranged for eligible service users who are interested in participating. Service users will be invited to identify a primary caregiver or a person of their choice to participate in the study and to be present at the home visit. To recruit a pragmatic and inclusive sample, service users who do not have an available caregiver will still have an opportunity to participate in the study.

At the home visit, the trial social worker will give verbal and written information on the study in isiXhosa. If the participant is interested in participating, the social worker will conduct the full capacity assessment, consent procedures, and baseline assessment. All procedures will be conducted privately. If the potential participant needs time to consider the information, a separate meeting will be scheduled within the following week. The subset of eligible but declining participants will be contacted by telephone by the study coordinator to invite them to participate in the qualitative interview.

#### Consent

Service user capacity to consent will be assessed by the trial social worker, who will be trained by an isiXhosa-speaking psychiatrist. We will use a modified capacity assessment form shown to be feasible in other LMIC settings [26, 27]. Participants assessed not to have decision-making capacity will be excluded, as well as their caregiver. The trial social worker will obtain informed consent for service users with capacity and their caregivers. For participants who are unable to write, a thumb impression will be recorded, along with a witness’ signature. To compensate for their time, participants will be provided with a R150 grocery voucher at each assessment time point. Written informed consent for ASWs, nurses, clinic managers, and declining individuals participating in qualitative interviews will be obtained by the study coordinator.

#### Sample size

We will aim to recruit a total of 100 service users and 100 linked caregivers. Assuming 20% attrition, ~40 service users and ~40 caregivers will receive the intervention and each recovery group will have a mean size of 11.4 participants (5–6 service users and 5–6 caregivers). We envisage these are sufficient numbers to adequately assess the acceptability and feasibility of the intervention (Objective 1.a), whilst accounting for potential differences in experience due to variations between clinic catchment areas, e.g. in socio-economic status, accessibility of venue, and community resources.

For the validation of outcome measures (Objective 2.a), we estimate that our final sample size of *n*=80 service users will give >90% power to detect correlations of ≥0.4 and 70% power to detect a standardised effect size of 0.28 for internal sensitivity to change at 2 months compared to baseline. The effect size of 0.28 is based on a mean change of 5 from baseline to follow-up, with a standard deviation of 15 and an intraclass correlation of 0.05. As this is a feasibility study, it is not powered to detect differences in outcome measures between treatment arms (Objective 3.a). We anticipate a total of approximately *n*=4 ASWs, *n*=7 service user facilitators, and *n*=7 caregiver facilitators will be sufficient to make an exploratory assessment of facilitator competence (Objective 1.b).

### Interventions

#### Treatment as usual (TAU)

TAU consists of treatment at the clinic, delivered mainly by psychiatric nurses. A general physician typically provides care at each clinic on an approximately weekly basis, but this varies across clinics. Frequency of attendance is determined by clinical need, but monthly appointments are the norm. Treatment includes prescription of anti-psychotic medication, symptom checking, and basic psychoeducation. Nurses can refer to the physician within the clinic, if available, for complex medication or clinical needs. Further referral can be made by the psychiatric nurse or physician to inpatient care at local hospitals that provide treatment for psychiatric emergencies. Participants will be able to discontinue TAU or recovery groups at their request. No concomitant care or interventions will be prohibited.

#### Recovery groups: design and overview

A theory of change map (Additional file 1) was developed during the formative phase as a model for explaining how the PRIZE intervention might facilitate and support psychosocial recovery [28]. The PRIZE theory of change map indicates (i) Participant outcomes: aspects of recovery deemed to be both important to service users and caregivers, and achievable through recovery groups, and (ii) Preconditions: aspects of participant engagement with, and delivery of, recovery groups theorised as necessary to attain the desired outcomes. The recovery group intervention, training, and supervision was designed to achieve the theory of change preconditions and outcomes. The intervention arm will comprise seven recovery groups, each linked to a clinic catchment area and comprising both service users and caregiver participants. Recovery groups will be delivered in two phases: a 2-month phase facilitated by an auxiliary social worker, then a 3-month peer-led phase.

##### Auxiliary social worker facilitator phase

Indlela Mental Health is a charitable organization offering community-based psychosocial support for people with intellectual disabilities in Nelson Mandela Bay Metropolitan district.

Two ASWs currently working at Indlela Mental Health will facilitate the recovery groups. ASWs have completed a 1-year accredited diploma. Two assistant facilitators, who have a social work degree but no prior work experience, will support the facilitation. Assistant facilitators are not currently employed at Indlela Mental Health but will receive a stipend. Each pair will facilitate three or four groups. ASWs and assistant facilitators will be trained over a total of 5 days by an adult education specialist, the study investigators, the isiXhosa-speaking study coordinator, and a person with lived experience of psychosis and experience of delivering peer support. Three training days will be held in advance of groups starting. Four subsequent half-day training sessions will be held on a fortnightly basis, staggered between group sessions in an apprenticeship model [29]. Training will cover recovery group values, facilitation skills, session content, supervision processes, and when to request support. Training will follow a manual and use participatory and experiential learning methods including discussions, activities, and role plays.

Participants allocated to recovery groups will receive a home visit or telephone call from an ASW in advance of the group sessions. At this contact, the ASW will give more details on the aims, structure, and potential benefits of the groups. This is distinct from the earlier recruitment home visit. Groups will follow a staggered start pattern with at least 2 weeks between the first and second groups commencing. Sessions will last 2 h and will be held in community centres within walking distance from participants’ homes. Attendance lists for each group meeting will be taken. Each session will cover a specific topic (see Table 1).

**Table 1:** Auxiliary social worker facilitated sessions

PRIZE recovery groups: Auxiliary social worker facilitated sessions	
1. Introduction to the recovery group	
2. Understanding my mental health	
3. Building self-esteem	
4. Recovery planning 1: My personal recovery plan	
5. Recovery planning 2: When things aren’t going well	
6. Recovery planning 3: Dreams and goals	
7. Thinking about money	
8. Healthy relationships	
9. Celebrating our journey so far and next steps together	

Sessions will be manualised and follow a standard format which guides the topic, approach, and group exercises. The session guides do not include detailed information. Instead, it is envisioned that group participants will generate much of the session content through their own ideas and experiences. Each session will begin with a check-in for separate service user and caregiver groups. The subsequent session activities will involve service users and caregivers together: sharing ideas in group discussion, e.g. on problems experienced, coping strategies; group problem solving; information provision/ signposting to services; and informal socialising. Recovery stories, which are anonymised composite narratives derived from qualitative data collected from service users in the formative phase, will be used as a starting point for most discussions. The first session will include signing of a group confidentiality agreement. Refreshments will be provided. After the first delivery of each of the ASW-facilitated sessions, a meeting will be held with ASW and investigators to gather initial feedback on their experience of delivery. Suggested minor iterative changes to session content, structure or documentation will be made as required in advance of subsequent delivery of that session in other groups. Substantial changes are not anticipated, meaning that intervention delivery should be comparable between groups.

ASWs will be supervised by a social worker, employed by Indlela Mental Health. This social worker will receive a half-day supervisor training in addition to participating in the ASW facilitator training. Supervision will comprise (i) A weekly group debrief with the ASWs, assistant facilitators and the social worker and (ii) A monthly observed session, at which the social worker will complete a GroupAct assessment for each facilitator and give immediate feedback [30]. The GroupAct tool assesses group facilitation skills by scoring on unhelpful or potentially harmful behaviours, and basic and advanced helping skills. The GroupAct results will be used to guide subsequent training sessions. Additional sessions will be observed if serious or persistent problems are identified. This use of the GroupACT as a supervision aid is conceived as integral to the intervention design, as distinct from its use in the process evaluation (see below). Additionally (iii) the social worker will provide ad hoc support to facilitators via telephone to address any arising issues such as safety concerns.

##### Peer facilitator phase

At weeks 4–5 of the ASW-facilitated phase, two peer facilitators will be identified from each group through self and group member nominations. There will be flexibility as to the configuration (one service user facilitator and one caregiver facilitator or two of either). Further variations may be considered depending on the wishes of the group (for example, three peer facilitators) and group problem solving will be used to address emerging challenges (for example, if no candidates are forthcoming). Peer facilitators will not be renumerated for their role. Self-organization and self-determination are core values of the recovery groups and the transition to the peer-led format will be directed by the groups. The peer facilitators (~14 in total) will then be trained over four half-day sessions by the ASWs, the isiXhosa-speaking study coordinator, and study investigators. Training will cover facilitation skills, session structure, and when to request support. Training will follow a manual and use participatory and experiential learning methods. Peer facilitators will be given a two-page universal session outline, including problem-solving steps, in isiXhosa.

ASWs will supervise the peer facilitators, through the following approaches: (i) ASWs will observe the first two peer-led sessions for each group, then attend groups monthly. At these monthly visits, ASWs will complete a GroupAct assessment of facilitation skills for each peer facilitator and provide immediate supportive feedback. The GroupAct results will also be used to guide subsequent training sessions. Additional sessions will be observed if serious or persistent problems are identified. (ii) ASWs will have a telephone debrief with each peer facilitator after every session (unless observed) and (iii) Group supervision with all peer facilitators will be held on a fortnightly basis, to share experiences and discuss and problem-solve any emerging issues.

#### Strategies to increase participation in recovery groups

To promote group participation, ASWs will contact each participant via their preferred method (WhatsApp/ text message or phone call) the day prior to each ASW and peer-led session. ASWs will provide group members with a reminder card for the following week’s session. Facilitators will keep an attendance register for each session and, in line with the empowering purpose of the group, encourage participants to consider ways of improving attendance if it becomes problematic. ASWs will contact non-attending group members via WhatsApp/ text message or phone call to check their wellbeing and encourage them to attend the following week.

### Assignment of interventions

The randomisation code will be generated by an independent statistician using permuted block randomisation. The randomisation will be stratified by clinic catchment area. The trial social worker will supply the study coordinator with the details of all recruited participants, including study ID. For each recruited participant, the study coordinator will determine the allocation code using the Redcap randomisation module. They will then inform the participant of the trial arm to which they have been allocated (TAU or TAU + recovery groups) by telephone. The trial social worker and assessor will be informed of the participant ID number prior to baseline data collection, but will be masked to allocation status. At the 5-month study endpoint, assessors will be asked to report whether they have been unmasked to allocation status. Data analysts will also be masked to allocation status. Due to the nature of the intervention, it is not possible to mask participants or interventionists (ASWs or social workers).

### Outcomes and data collection

#### Process evaluation methods

##### Overview

The process evaluation will address Objectives 1.a (intervention acceptability and feasibility) and 1b (facilitator competence). One or more process indicators spanning qualitative and quantitative data were selected for each precondition on the theory of change (see Table 2 and Additional file 1). For example, to assess the precondition “P6: Peers have interest and willingness to be facilitators”, in-depth interviews (IDIs) with peers will include a prompt on barriers and motivation to taking on the facilitator role (qualitative) and the number of peer facilitators identified for each group will be recorded (quantitative).

**Table 2:** PRIZE process evaluation preconditions, indicators, and data types

Pre-conditions for intervention to achieve desired outcomes	Indicator/s	Data type/s
P1: Service users and caregivers are identified and want to attend group sessions	Number of service users and caregivers consenting to participate	Quantitative
	Reasons for declining participation	Quantitative & qualitative (decliners IDIs)
P2: ASWs attend (ongoing) training	Number/% training sessions attended	Quantitative
P3: Social worker supervises ASWs	Number of supervision sessions attended	Quantitative
	Supervision perceived to be adequate	Qualitative (ASW IDIs)
P4: ASWs have skills to successfully facilitate groups	GroupACT scores from observed group sessions	Quantitative (GroupACT)
	(Self-)Perception of facilitation skills and competence	Qualitative (service user, caregiver & ASW IDIs)
P5: ASWs remind peers to attend	Number/% participants with attempted reminder, e.g. call attempted, message sent	Quantitative
	Number/% participants with reminder successfully conveyed, e.g. message read, participant spoken to	Quantitative
P6: Peers have interest and willingness to be facilitators	Two peer facilitators identified for each group	Quantitative
	Barriers and motivators to taking peer facilitator role	Qualitative (service users and caregiver IDIs)
P7: ASWs support peer facilitators	Number of peer-facilitated sessions shadowed by ASW	Quantitative
	Perception of adequacy of support received	Qualitative (peer facilitator IDIs)
P8: ASWs refer participants to services in line with recovery plan	Number of referrals made to Indlela Mental Health	Quantitative
	% of referrals to Indlela Mental Health resulting in service contact	Quantitative
	Perception of whether referrals are in line with recovery plan	Qualitative (service user IDIs)
P9: Peers have sense of group belonging and ownership	Perception of belonging and ownership	Qualitative (service user, caregiver & ASW IDIs)
P10: Peers attend sessions regularly	% attendance at sessions	Quantitative
P11: Peers share personal experiences and coping strategies	Perception of degree of sharing experiences/ strategies	Qualitative (service user, caregiver & ASW IDIs)
P12: Peers develop personal recovery plan	Perception of how engaged participants are in recovery planning	Qualitative (service user, caregiver & ASW IDIs)
P13: Peers shape group focus to their priorities	Number of external speakers identified/ invited to group	Quantitative
	Perception of degree of shaping to peer priorities	Qualitative (service user, caregiver & ASW IDIs)
P14: Peers solve problems to work towards recovery	All planned ASWs sessions completed	Quantitative (fidelity checklist)
	Perception of usefulness of ideas and information for recovery	Qualitative (service user & caregiver IDIs)
P15: Caregivers develop strategies to support their relative	(Self-)Perception of caregiver strategies and skills	Qualitative (service user & caregiver IDIs)
P16: All peers contribute to running of group	Perception of peer contribution	Qualitative (service user & caregiver IDIs)
P17: Peer facilitators attend (ongoing) training	Number/% peer training sessions attended	Quantitative
P18: Peer facilitators have skills to successfully facilitate groups	GroupACT scores from observed group session	Quantitative (GroupACT)
	(Self-)Perception of facilitation skills and competence	Qualitative (service user, caregiver & ASW IDIs)

##### Qualitative data collection and analysis (process evaluation)

Table 3 outlines qualitative data collection participants, format, timing, and key topics. Three to five IDIs with service users and caregivers declining to participate in the study will be conducted at baseline, covering unmet needs and barriers to participation. ~30 IDIs will be conducted at 5 months after recruitment with service users, caregivers, ASWs, the social worker supervisor, and mental health nurses, to assess the acceptability and feasibility of peer-led groups. Topics will include acceptability of the group format, perceived usefulness of group problem solving for recovery, perceptions of ASW and peer facilitators, and perceived barriers and facilitators of participation and impact (see Table 3). Focus group discussions (FGDs) will be held with peer facilitators (separate groups for service users and caregivers) covering adequacy of training and supervision and self-perception of facilitation skills. IDIs and FGDs will be conducted in isiXhosa in participants’ homes or the clinic in a private space by a qualitative researcher whose first language is isiXhosa and will be audio recorded. All participants will be provided with information about the purpose of the interview and have written informed consent taken by researcher. For each set of IDIs, the first 2–3 interviews will be rapidly transcribed, translated, and reviewed by investigators, prior to conducting further IDIs.

**Table 3:** Qualitative data collection participants and topics

Participant type	Number & data collection	Purposive sampling	Key topics
Caregivers and service users declining to participate in pilot	3–5 IDIs	Gender; reason for declining question	Needs; perceived usefulness of intervention; barriers to participation
Service user and caregiver recovery group participants	10–12 service user IDIs & 10–12 caregiver IDIs	Gender, group/ clinic; number of sessions attended	Acceptability of recovery groups (including group format, frequency, location, perceptions of ASW and peer facilitators); peer contribution to groups; usefulness of group problem solving for recovery; appropriateness/usefulness of referrals; met and unmet needs; and perceived barriers and facilitators of participation and impact
Peer facilitators in intervention arm	1 FGD service users, 1 FGD caregivers (both with all facilitators)	n/a	Adequacy of training and supervision; self-perception of facilitation skills/ competence; impact of peer facilitator role on recovery; sustainability of role
Auxiliary social worker facilitators	2 IDIs	n/a	Adequacy of training and supervision; self-perception of facilitation skills/ competence; perception of peer contribution to groups; feasibility/ sustainability of role
Social worker supervisors	1 IDI	n/a	Adequacy of training and supervision; perception of ASW facilitation skills/ competence; feasibility/ sustainability of role
Facility staff (Psychiatric nurses & clinic managers)	3–5 IDIs	Clinic	Feasibility/ sustainability and utility of groups
Control arm participants	3–5 IDIs	Clinic; response to contamination question	Awareness of group existence and aims; perceived impact on mental health

Thematic analysis using an inductive approach will be conducted for IDIs and FGDs. After an initial familiarization process, a minimum of two transcripts from each participant group will be coded by two project staff using NVivo 12 [31] and an initial coding framework jointly developed. They will then meet after coding every 5–10 transcripts to assess coding agreement. Coding discrepancies will be resolved through discussions with a third member of the project team. Codes will be collated into themes and subthemes, and codes and themes will be refined as coding progresses [32].

##### Quantitative data collection and analysis (process evaluation)

To address Objective 1.a (intervention feasibility), the following set of process indicators will be enumerated (see Table 2): (i) number of sessions attended by group participants, (ii) number of training and supervision sessions attended by facilitators, (iii) number of peer facilitators identified for each group, (iv) number of peer-led sessions shadowed by ASW, (v) proportion of session reminders attempted and conveyed by ASW, and (vi) Number of referrals made by ASW to Indlela Mental Health and proportion resulting in service contact. A descriptive analysis of all process indicators will be undertaken (numbers and proportions and/or means and standard deviations and/or medians and interquartile range).

To address Objective 1.b (facilitator competence), group facilitation skills of ASW and peer facilitator will be assessed with the GroupAct [33]. For the purposes of the process evaluation, the study coordinator will act as a rater/s following training. The study coordinator will observe group sessions to complete GroupAct assessments on weeks 1 and 8 of ASW-facilitated intervention delivery and weeks 1 and 8 of peer-facilitated intervention delivery. A descriptive analysis of GroupAct data will be completed. For each time point, we will generate means and standard errors for each item (including all ASW or peer facilitators) and mean item scores for each facilitator (across all items). Summary means will be generated for each time point.

In addition, for each ASW-facilitated session, facilitators will complete a short fidelity checklist indicating whether the core session components were carried out as planned. When sessions are observed by a social worker, or study coordinator, the observer will independently complete the fidelity checklist and agreement will be assessed. A descriptive analysis of fidelity checklist scores will be undertaken (e.g. proportion of sessions completing each item). All analysis of quantitative process data will be completed using Stata 15.0 [34]. For preconditions with qualitative and quantitative indicators, data will be independently analysed, then data sources compared for areas of convergence and divergence.

#### Preliminary outcome evaluation methods

##### Overview

The outcome evaluation will address Objective 3.a. (potential effectiveness). To ensure that the outcome evaluation measured potential intervention effects of importance to service users, one or more outcome measure was selected for each desired outcome on the theory of change (see Table 4).

**Table 4:** PRIZE desired outcomes and measure/s

Desired outcome	Measure/s
**Service users**	
T1: Feeling positive and hopeful	RAS-DS
T2: Feeling good about myself	ISMI, RAS-DS
T3: Feeling that my mind is working well	CANSAS, WHODAS, RAS-DS
T4: Having the personal relationships I want	CANSAS, WHODAS, RAS-DS
T5: Being respected & involved in my community	WHODAS, ISMI, Perception of respect and value
T6: Having meaning in life & making a contribution	RAS-DS
T7: Being independent	CANSAS, WHODAS-12
T8: Keeping healthy (good diet, no alcohol)	CANSAS, AUDIT-C
T9: Knowing what keeps me well and how to access it	RAS-DS, Health service use, Anti-psychotic Medication Adherence
**Caregiver**	
T10: Minimising the burden of caregiving	IEQ

*RAS-DS* Recovery Assessment Scale- Domains and Stages, *ISMI* Internalized Stigma of Mental Illness Scale, *CANSAS* Camberwell Assessment of Need- Short Appraisal Schedule, *WHODAS-12* 12-item World Health Organisation Disability Assessment Schedule, *AUDIT-C* Alcohol Use Disorders Identification Test- Consumption, *IEQ* Involvement Evaluation Questionnaire

##### Quantitative data collection (outcome evaluation)

The following service user outcome measures will be assessed in both trial arms: functioning (self- and proxy-rated 12-item WHO Disability Assessment Schedule (WHODAS) [35, 36]), personal recovery (Recovery Assessment Scale-Domains and Stages (RAS-DS) [37]), unmet needs (Camberwell Assessment of Need Short Assessment Schedule), internalised stigma (Internalized Stigma of Mental Illness Scale (ISMI) [38]), perception of respect and value (two newly developed questions based on formative work), alcohol use (Alcohol Use Disorders Identification Test- Consumption (AUDIT-C)) [39], health service use (bespoke questions), relapse, anti-psychotic medication adherence (5-point ordinal scale). The caregiver outcome is caregiver burden (caregiving consequences of the Involvement Evaluation Questionnaire (IEQ) [40]) (Table 5). Support for recovery (Brief INSPIRE) will be assessed in intervention arm participants only, in relation to their ASW facilitator (2-month endpoint), peer facilitator (5-month endpoint), and psychiatric nurse (baseline, 2- and 5-month endpoints). All instruments have been translated into isiXhosa and back translated to English to check for semantic equivalence. In addition, cognitive interviewing has been carried out for the WHODAS, CANSAS, and RAS-DS to detect difficulties with understanding of items and response categories and translation, and amendments made accordingly to ensure content validity.

**Table 5:** Quantitative outcome measures

Outcome	Measure	Details
Disability	Self-rated and proxy-rated 12-item WHO Disability Assessment Schedule (WHODAS) 2.0	The 12-item WHODAS 2.0 is a generic instrument for assessing disability relating to any health condition across cultures. It has been used previously in South Africa for people with severe mental illness [24]. Item-response theory-based scoring will be used to convert scores to a 0–100 scale [36]. The WHODAS proxy version has the same properties as the self-rated version but has been designed to be answered by a caregiver, relative or friend [36].
Recovery	Recovery Assessment Scale- Domains and Stages (RAS-DS)	The RAS-DS is a self-report measure of mental health recovery [37]. It includes 38 items clustered into four domains of recovery: functional recovery (“Doing things I value”); personal recovery (“Looking forward”); clinical recovery (“Mastering my illness”); and social recovery (“Connecting and belonging”). Each item is rated on a 4-point scale from 1 = “untrue” to 4 = “completely true”. “Percentage scores” are calculated for each domain and an overall score higher score represents more advanced levels of mental health recovery. It has been used previously in clinical settings in South Africa.
Unmet needs	Camberwell Assessment of Need – Short Appraisal Scale (CANSAS)	The CANSAS includes a list of 22 areas considered as potentially important needs for individuals living with mental illness. Each item is rated as either an “unmet need”; “met need” or “no need”. Percentage of unmet needs will be calculated based on number of unmet needs divided by total number of needs identified (unmet needs plus met needs). The scale has been previously used in South Africa [24].
Support for recovery	Brief INSPIRE	The brief INSPIRE assesses recovery support from a worker and has 5 items, each rated 0 “not at all” to 4 “very much”. Responses can be converted to a total score, ranging from 0 (low recovery support) to 100 (high recovery support) [41].
Internalised stigma	Internalized Stigma of Mental Illness (ISMI) Scale	The ISMI-R is a 29-item questionnaire assessing internalised stigma covering four subscales: “alienation”; “stereotype endorsement”; “perceived discrimination”; and “social withdrawal”. Items are scored on a 4-point Likert scale, from strongly disagree to strongly agree. Total scores are calculated by summing the items [38]. The scale has been previously used in South Africa [24].
Perception of respect and value	2 bespoke questions	Two questions “I feel valued and respected by my family” and “I feel valued and respected by my community” will be rated on a 4-point Likert scale, from strongly disagree to strongly agree.
Alcohol use	Alcohol use disorders identification test consumption (AUDIT-C)	The AUDIT-C includes three questions on alcohol consumption, each rated on a 0 to 4 scale. A total score can be calculated. It has been widely used and shown to be useful for assessing alcohol use in the South African context [39].
Health service use	Bespoke questions	Questions include number, duration and reason for inpatient admissions; and number of consultations with different types of healthcare worker in the last 2 months.
Relapse	Questions on police contact & hospitalization	Relapse is defined as either of: • Inpatient admission for mental health of any duration (assessed as part of health service use) • Any type of police contact related to mental health
Anti-psychotic Medication adherence	1 question	We will use a 5-point nominal scale measuring frequency of anti-psychotic medication adherence
Caregiver burden	Caregiving consequences section of Involvement Evaluation Questionnaire (IEQ)	31-item questionnaire assessing aspects of burden for caregivers of people with severe mental illness. All items are scored on 5-point Likert scales (0 never to 4 always). Domain scores can be computed (tension & urging range 0 to 36; worrying & supervision range 0 to 24)

Quantitative data will be collected at baseline, 2 months, and 5 months post-intervention start (see Fig. 1) at the participant’s clinic or at their home, with the exception of the Brief INSPIRE. Socio-demographic data will be collected at baseline. To avoid unmasking assessors, the study coordinator will collect brief INSPIRE data by telephone from intervention arm participants. Study data will be collected and managed on Android tablets using REDCap (Research Electronic Data Capture) electronic data capture tools hosted at the South African MRC [42, 43]. REDCap is a secure, web-based software platform designed to support data capture for research studies, providing (1) an intuitive interface for validated data capture; (2) audit trails for tracking data manipulation and export procedures; (3) automated export procedures for seamless data downloads to common statistical packages; and (4) procedures for data integration and interoperability with external sources. Assessors will be trained to administer the instruments using interactive techniques including role plays. The study coordinator will oversee data collection. Attrition will be minimised through phone calls and/or text message reminders to attend data collection and in-person tracking.

##### Quantitative data analysis (outcome evaluation)

The analysis will be completed using Stata 15.0 [34]. Means and standard deviations (or medians with interquartile ranges, where appropriate) will be reported for continuous outcomes and raw counts (number, %) for categorical data. To estimate the potential effect of recovery groups at 2 and 5 months, quantitative outcomes, except Brief INSPIRE, will be compared between treatment arms, adjusting for baseline scores and other predictors, using linear mixed models for continuous variables and generalised linear mixed models for binary variables based on an intention-to-treat analysis; 95% confidence intervals, but not *p* values, will be reported. Adjustment will be made for the within-clinic correlation, and the intracluster correlation will be reported. We will use standard methods for dealing with single missing items from continuous scales, including taking the average across items, or imputing the item [36]. Missing data for other outcomes will not be imputed.

To assess differences in support for recovery between service providers, the paired *t*-test will be used to compare Brief INSPIRE scores amongst intervention arm participants between (i) ASW facilitator support at 2 months and peer facilitator support at 5 months; (ii) ASW facilitator support and psychiatric nurse support at 2 months; and (iii) peer facilitator support and psychiatric nurse support at 5 months. Further analysis may include a linear mixed effects model with scores at all follow-up visits as outcome and scores at the previous time point as predictor. Adjustment will be made for within-clinic correlation.

#### Assessment of trial procedures

To assess the validity of key outcome measures (Objective 2.a), convergent validity of baseline proxy-reported WHODAS and CANSAS will first be determined by calculating Pearson’s correlation coefficient (*r*). Internal and external responsiveness to change from baseline to 2 and 5 months will be calculated, using proxy-reported WHODAS as an external reference measure. These steps will be repeated for the RAS-DS, using proxy-reported WHODAS as an external reference, and for RAS-DS using CANSAS as an external reference measure. Cronbach’s Alpha will be reported as a measure of internal consistency of the measures. Exploratory Factor Analysis with varimax orthogonal rotation will be conducted to identify any possible factors which the measures may load on.

To assess the feasibility of trial procedures (Objective 2.b), a meeting will be held with ASWs, the social worker, and the study coordinator to gather feedback on the feasibility of the GroupAct and fidelity checklist, e.g., time taken to rate, ease of use, usefulness in providing immediate feedback and identifying training needs. Numbers of eligible participants at each clinic will be compared to predicted numbers (*n*=14) and the proportion consenting to participate will be calculated. The proportion of, and reasons for, participants lost to follow-up at 2 months and 5 months will be recorded. At the 5-month endpoint, all control arm participants will be assessed for contamination including awareness of recovery groups, using a single item administered as the last question on the outcome evaluation assessment. In addition, IDIs will be carried out with control arm participants to gain a more in-depth understanding of extent and means of awareness of intervention groups, and potential impact of exclusion from the groups on their wellbeing (see Table 3). Outcome measure means and standard deviations will be used to inform the sample size calculations for a definitive trial (Objective 2.c).

### Data management and trial oversight

A trial steering committee will provide oversight of study progress and participant safety. Detection and reporting of serious adverse events and protocol violations will be guided by dedicated standard operating procedures. Quantitative data will be uploaded daily to the MRC’s secure data storage in Cape Town, South Africa. All IDIs and FGD transcripts will be anonymised and identifiable only through a unique identification number. During transcription, the research assistant will omit any identifiable information included in the audio-recording. Participant details and locator information will be stored separately to transcripts in a password-protected file. Transcripts and audio files will be stored in password-protected files on study computers.

## Discussion

The PRIZE study will contribute to the sparse evidence on the acceptability and feasibility of peer-led and recovery-oriented interventions for people with psychosis in LMIC. By integrating the PRIZE intervention into the existing health and social care system in South Africa, we aim to understand the feasibility and acceptability of delivering this intervention in a real-world setting without a substantial injection of new resources. A key strength of the study is that the PRIZE recovery groups are genuinely recovery oriented; that is, the content and delivery of the groups is shaped by its members, rather than focused on achieving narrow or externally defined goals such as “becoming ‘normal’ and ‘independent’ of support and services” [44].

Another important strength is that the process and outcome evaluations are theoretically driven [45]. Both evaluations directly map onto a theory of change, documenting the causal assumptions underpinning the intervention, which itself was shaped by our in-depth formative work. The process evaluation will use mixed methods to provide a rich account of how the PRIZE recovery groups work in practice. Outcome measures were selected to assess only outcomes of key importance to service users. Outcomes initially mooted by the study team were excluded if they did not meet this criterion, e.g. symptom severity. Where existing suitable measures were not identified, bespoke questions (e.g. on the perception of respect and value by family and community) were added to the assessment battery.

An important limitation is that service users were not actively involved in design of the intervention and only minimally involved in training. This was due to logistical difficulties relating to the COVID pandemic and the absence of a local existing pool of peer supporters. Future research and implementation activities should prioritise involvement of people with lived experience of psychosis. Some features of the study design differ from procedures that would likely be followed in a real-world setting. For example, rather than simply exclude participants assessed not to have decision-making capacity, as well as their caregiver, such participants could be reassessed for inclusion later and included in a subsequent recovery group cohort.

The PRIZE recovery group design and delivery arrangements pose several potential challenges. First, despite joint workload planning between study investigators and Indlela Mental Health, it is possible the ASWs and the supervising social worker will not have sufficient time to attend all groups and training sessions on top of their existing workload. Second, service users and caregivers may not have time or interest to participate. Absence of an income generating component in an underserved population with high levels of unemployment may mean the groups do not directly meet participants’ most pressing needs [46, 47]. By making participation of caregivers optional, we hope to mitigate the impact of these potential barriers on service user attendance. Third, we might not identify participants willing to take the role of peer facilitator. If that occurs, we intend to use problem solving by recovery group members to identify potential solutions; more intensive support by ASWs may be one possible approach. Finally, future COVID outbreaks or other unforeseen events might make group meetings unfeasible. In this scenario, we will conduct a risk assessment and adaptations will be made where possible to continue in-person groups (e.g., socially distanced outdoor meetings). If this is not possible, we will consider other means of maintaining continuity and contact with peers without in-person contact (e.g., by phone). The feasibility and acceptability of any amendments to the format will be investigated using the process evaluation methods already planned. The PRIZE feasibility trial results, including whether such challenges emerge in practice and how they can be overcome, will help to refine the intervention in preparation for a definitive trial and subsequent larger-scale implementation.

## Supplementary Information

**Additional file 1.** 

## Data Availability

Data sharing is not applicable to this article as no datasets were generated or analysed during the current study.

## Abbreviations

ASW: Auxiliary social worker
AUDIT-C: Alcohol Use Disorders Identification Test-Consumption
CANSAS: Camberwell Short Assessment of Needs Schedule
FGD: Focus group discussion
HIC: High-income countries
IDI: In-depth interview
IEQ: Involvement Evaluation Questionnaire
ISMI: Internalized Stigma of Mental Illness scale
LMIC: Low- and middle-income countries
PRIZE: Peer-led recovery groups for people with psychosis in South Africa
RAS-DS: Recovery Assessment Scale- Domains and Stages
RedCAP: Research Electronic Data Capture
SAMRC: South African Medical Research Council
TAU: Treatment as usual
WHO: World Health Organization
WHODAS: World Health Organization Disability Assessment Schedule
