# Supplementary material for: Peer-led recovery groups for people with psychosis in South Africa (PRIZE): protocol for a randomised controlled feasibility trial
Source: Pilot Feasibility Stud. 2023 Feb 1;9:19. doi: 10.1186/s40814-022-01232-8 (PMC9890934; doi:10.1186/s40814-022-01232-8)
Supplement: Supplementary file 1 — Additional file 1. [file 40814_2022_1232_MOESM1_ESM.pptx]

## Slide 1
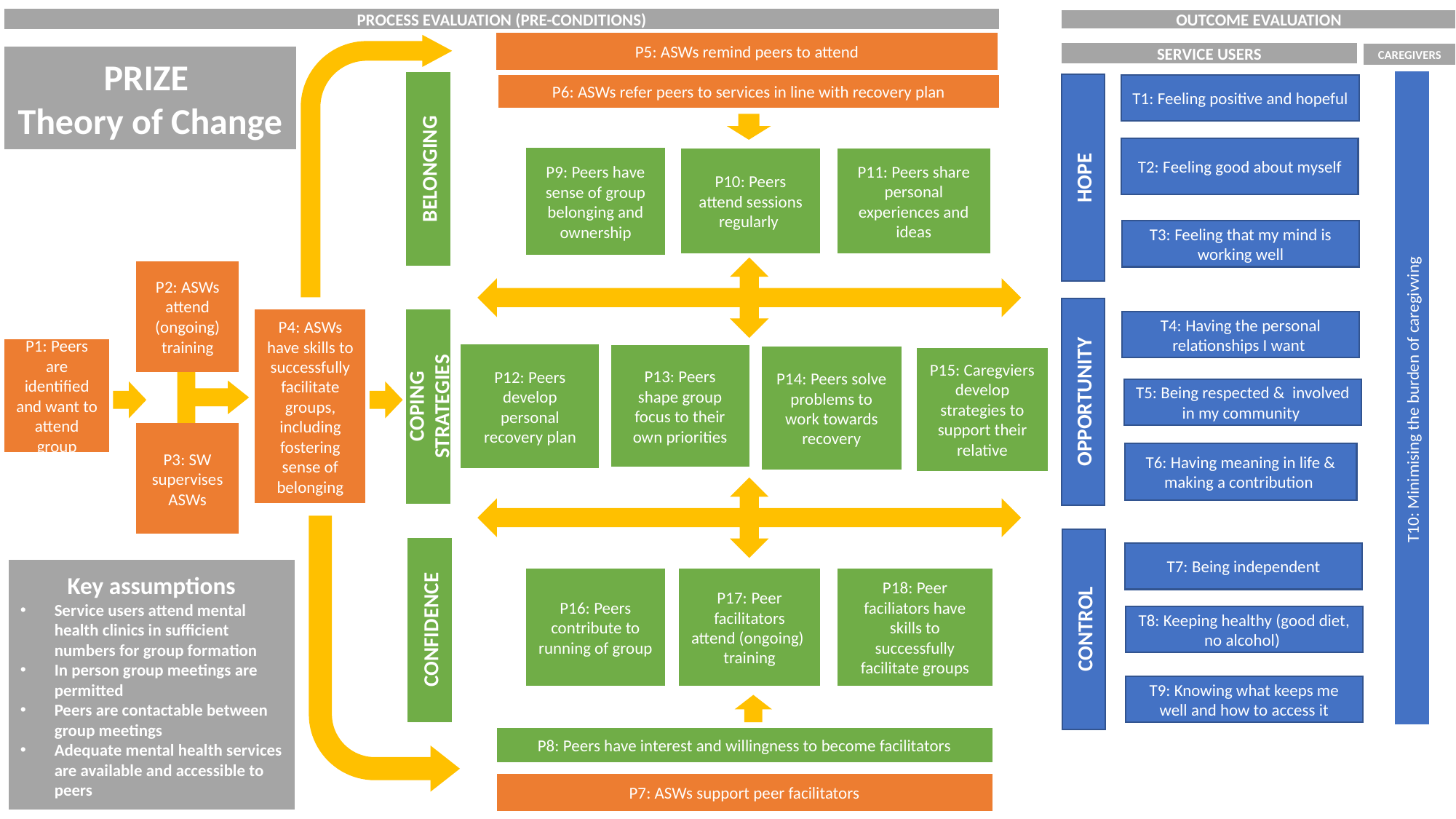

PROCESS EVALUATION (PRE-CONDITIONS)
OUTCOME EVALUATION
P5: ASWs remind peers to attend
SERVICE USERS
CAREGIVERS
PRIZE
Theory of Change
T1: Feeling positive and hopeful
P6: ASWs refer peers to services in line with recovery plan
T2: Feeling good about myself
BELONGING
P9: Peers have sense of group belonging and ownership
P10: Peers attend sessions regularly
P11: Peers share personal experiences and ideas
HOPE
T3: Feeling that my mind is working well
P2: ASWs attend (ongoing) training
P4: ASWs have skills to successfully facilitate groups, including fostering sense of belonging
T4: Having the personal relationships I want
P1: Peers are identified and want to attend group
P12: Peers develop personal recovery plan
P13: Peers shape group focus to their own priorities
P14: Peers solve problems to work towards recovery
P15: Caregviers develop strategies to support their relative
T5: Being respected & involved in my community
OPPORTUNITY
T10: Minimising the burden of caregivving
COPING STRATEGIES
P3: SW supervises ASWs
T6: Having meaning in life & making a contribution
T7: Being independent
Key assumptions
Service users attend mental health clinics in sufficient numbers for group formation
In person group meetings are permitted
Peers are contactable between group meetings
Adequate mental health services are available and accessible to peers
P16: Peers contribute to running of group
P17: Peer facilitators attend (ongoing) training
P18: Peer faciliators have skills to successfully facilitate groups
T8: Keeping healthy (good diet, no alcohol)
CONTROL
CONFIDENCE
T9: Knowing what keeps me well and how to access it
P8: Peers have interest and willingness to become facilitators
P7: ASWs support peer facilitators
